# Health policies in long-term care facilities for older adults: A systematic review of textual evidence

**DOI:** 10.1016/j.mex.2026.103798

**Published:** 2026-01-13

**Authors:** Florbela Bia, Matheus Kirton dos Anjos, Zaida Charepe, Cristina Marques-Vieira

**Affiliations:** a Departamento de Enfermagem, Universidade de Évora, Évora 7000-811, Portugal; b PhD Student, Faculty of Health Sciences and Nursing, Center for Interdisciplinary Research in Health (CIIS), Universidade Católica Portuguesa, Lisbon, Portugal; c Faculty of Health Sciences and Nursing, Center for Interdisciplinary Research in Health (CIIS), Universidade Católica Portuguesa, Lisbon, Portugal; d Nursing School of University of Lisbon (ESEUL), Centre for Research, Innovation and Development in Nursing (CIDNUR), University of Lisbon, Lisbon, Portugal; e Center for Interdisciplinary Research in Health (CIIS), Universidade Católica Portuguesa, Lisbon, Portugal

**Keywords:** Long-term care facilities, Healthcare policy, Aged, Health facilities: long-term care, Skilled Nursing Facilities

## Abstract

**Background:**

Health polices in residential care settings for older adults are essential to align housing and care provision with the needs of ageing populations, ensuring safety, participation, and quality of life. Despite their importance, evidence regarding policy implementation in long-term care (LTC) facilities remains fragmented and inconsistent.

**Objective:**

This protocol describes an original method for synthesizing health policy documents using the Joanna Briggs Institute (JBI) framework for textual evidence. It aims to identify, analyze, and integrate policy evidence related to LTC facilities for older adults.

**Methods:**

Following JBI methodological guidance for systematic reviews of text and opinion, this protocol employs the PICo framework to define inclusion criteria and a three-step search strategy. Searches will be conducted in MEDLINE, CINAHL Complete®, and the Virtual Health Library (BVS). Eligible documents include laws, regulations, policy frameworks, and technical guidelines addressing long-term care within publicly regulated systems. Data extraction and quality appraisal will be independently performed by two reviewers using JBI instruments.

**Expected Results:**

The review will synthesize existing polices, highlighting their characteristics, implementation strategies, and outcomes. By applying a transparent and replicable JBI-based method, this protocol supports the production of high-quality evidence to inform equitable and effective governance in LTC facilities

## Graphical abstract

The image included in the graphical abstract was generated with OpenAI’s ChatGPT from an author-written prompt; the content was reviewed by the authors; no real individuals are depicted.

**Figure:**
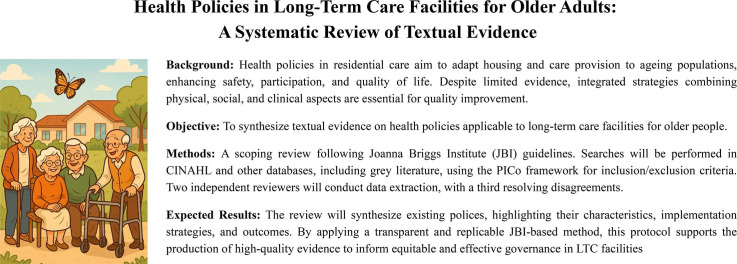

## Specifications table

TABLE 1. Specifications for the Systematic Review Protocol.

**Table:** 

**Subject area**	Medicine and Dentistry
**More specific subject area**	Clinical Reasoning in Nursing
**Name of your protocol**	Health Policies in Long-Term Care Facilities for Older Adults: A Systematic Review of Textual Evidence
**Reagents/tools**	N/A
**Experimental design**	This systematic review of textual evidence aims to synthesize the available textual evidence on health policies applicable to Long-term Care Facilities for Older People.
**Trial registration**	PROSPERO registration number: CRD420251132804.
**Ethics**	This investigation constitutes a systematic review and, as such, does not entail direct experimentation involving human or animal participants, nor does it include the collection of data from social media platforms. Consequently, ethical approval or informed consent was not required. All data examined were derived from previously published research that had already been subjected to the relevant ethical evaluation procedures. This review protocol is founded on ethical principles that ensure scientific integrity and credibility [[1], [2], [3]]. A review must accurately reproduce the content and meaning of the consulted sources [1]. The proper attribution of authorship, as highlighted by Plevris [2], constitutes an essential ethical obligation. Furthermore, the transparency and traceability of information are indispensable for the verification and validation of results [3]. Data availability: All data are derived from publicly available sources.
**Value of the Protocol**	This protocol presents an innovative use of the Joanna Briggs Institute (JBI) methodology to systemically synthesise health policy documents. By extending JBI’s textual evidence framework to the analysis of long-term care policy, it introduces a replicable method for integrating normative evidence into healthcare research. ⦁ Provides a structured framework to guide research on health policies in Long-term Care Facilities for Older Adults. ⦁ Ensures methodological rigor and transparency through adherence to JBI and PRISMA standards. ⦁ Facilitates methodological replication and supports future application of structured approaches to health policy analysis.

## Background

The global demographic transition towards an ageing population presents unprecedented challenges for health care systems worldwide. With the number of individuals aged 65 and older expected to nearly double by 2060, reaching approximately 94,7 million in the United States, the demand for long-term care (LTC) services has intensified significantly [4]. LTC facilities, including nursing homes and other residential institutions, form an integral part of the health care continuum for older adults who require ongoing assistance with activities of daily living and medical support [5].

Health policies that govern these residential care settings influence the organization and delivery of care, including quality standards, safety procedures, staffing requirements and infection control measures. Such policies encompass a broad range of regulatory frameworks, clinical guidelines and quality assurance mechanisms that collectively shape the care environment in long-term care facilities [6]. Implementing health polices in LTC facilities is complex, involving interactions among national, regional and local regulations that vary considerably across jurisdictions [7].

This variability highlights the need for a systematic examination of the existing policy frameworks that regulate LTC environments. Despite the recognized importance of effective policy implementation, few studies have comprehensively analyzed how these frameworks are structed an applied within residential care contexts [8]. Previous reviews have primarily focused on clinical or organizational aspects, often overlooking the normative and textual dimensions that underpin the governance of LTC systems.

To address this gap, the present protocol applies the Joanna Briggs Institute (JBI) methodology for textual evidence synthesis to policy and regulatory documents. This approach enables the systematic identification, analysis an interpretation of policies guiding long-term care for older adults. By providing a structured, transparent and replicable methodological process, the protocol offers a basis for consistent evidence synthesis in health policy research related to long-term care facilities.

Existing reviews on long-term care facilities have predominantly focused on clinical outcomes, staffing levels, quality indicators, or organizational performance. In contrast, this study adopts a policy-oriented perspective by systematically synthesizing normative and regulatory documents that govern residential long-term care for older adults. By applying the Joanna Briggs Institute (JBI) methodology for textual evidence, this protocol treats health policies and regulatory frameworks as primary sources of evidence rather than contextual background. This approach enables a structured, transparent, and replicable synthesis of policy texts across different jurisdictions, addressing a methodological gap in the literature and contributing to the advancement of evidence-informed health policy analysis in long-term care settings.

## Description of protocol

### Materials and methods

This systematic review protocol was developed in accordance with the Joanna Briggs Institute (JBI) methodology for systematic reviews of textual evidence, including policy and guideline documents [9,10]. The JBI Checklist for Policy and Guideline Documents will be used for critical appraisal, with data extraction and quality assessment conducted independently by two reviewers. Any disagreements will be resolved through discussion or consultation with a third reviewer. All methodological procedures are designed to be transparent, replicable, and adaptable to similar policy-oriented evidence syntheses.

The protocol follows the Preferred Reporting Items for Systematic Reviews and Meta-Analyses (PRISMA) guidelines and was prospectively registered in the International Prospective Register of Systematic Reviews (PROSPERO; registration number CRD420251132804). It was initially developed in July 2025 and amended in November 2025, with completion of the systematic review of textual evidence anticipated by December 2025.

To ensure methodological robustness, only peer-reviewed scientific literature was used to support the theoretical and methodological rationale of this protocol. Institutional and policy documents are considered exclusively as objects of analysis within the scope of the textual evidence synthesis.

## Eligibility criteria

• Population

This review will include documents addressing older adults residing in long-term care settings (e.g., nursing homes and assisted living facilities). Eligible sources will comprise official and normative documents, such as laws, regulations, policy frameworks, guidelines, strategic plans, and technical reports, addressing health and public policies for residential care of older adults or analogous institutions.

Only materials issued by recognized authorities (e.g., governmental bodies, the World Health Organization [WHO], the Organisation for Economic Co-operation and Development [OECD], professional associations, and regulatory agencies) will be considered. The scope will focus on institutional long-term care settings in Portugal and in countries with comparable health systems, namely publicly regulated institutions within European countries and OECD countries with universal health coverage and analogous policy frameworks.

Included documents must contain textual or normative content, exclude original empirical data, and relate to institutions providing continuous, 24-hour nursing care. Publications written in english, spanish, or portuguese will be eligible.

• Intervention

The review will include studies that focus on Long-term care; Older adult care; Healthcare policy

• Comparison

This review does not have any comparators.

• Primary Outcomes

The interpretative synthesis of these categories will seek to identify consensus, divergences and gaps in existing policies, as well as the evolution of institutional discourses over the last ten years. Where relevant, representative citations of the documents will be used to illustrate the analyses, ensuring fidelity to the original source.

• Additional outcomes

Additional outcomes include resident rights and safeguarding, equity provisions, inspection and enforcement mechanisms, data reporting and quality improvement, and care models/service packages. Extracted from official policy or regulatory documents. No measurement instruments; time point = publication/revision year. Effect measure not applicable; synthesis will be descriptive and thematic.

• Study Design

Only nonrandomized study types will be considered for inclusion. This study will prioritise grey literature concerning health policies in residential care facilities for older adults, such as technical documents, internal reports, procedural manuals, governmental and institutional papers, policy briefs, and working papers.

The search strategy will involve institutional and organizational websites, Google Scholar, and databases such as CINAHL Complete®, MEDLINE and the Virtual Health Library (VHL).

• Context

The review will include residential care environments for older adults, such as nursing homes, long-term care facilities (LTCF), assisted living residences, and equivalent institutions with public regulation. Eligible contexts are European countries, OECD countries with universal health systems, with structured and comparable long-term care policies.

Only official, institutional, and normative documents will be included. Documents must explicitly address the organization, governance, financing, staffing, or quality of care in residential care settings for older adults.

Documents relating to hospital, community, or home-based care contexts will be excluded, as will those focusing on caregivers or professionals without direct reference to older residents. Opinion papers, editorials, and commentaries lacking regulatory or normative significance will not be considered, nor will reports limited to biomedical aspects or to generic health policies that do not specifically address institutional care. Documents involving primary empirical research, or those of a purely administrative, procedural, or technical nature without a strategic or policy orientation, will be excluded. Informal or unregulated care settings, countries with predominantly private or non-comparable long-term care models, and unverified or unofficial sources will likewise not be included. Studies conducted in hospital wards, long-term care units, or short and medium-stay facilities will also be excluded.

## Search strategy

The search strategy will involve conducting a comprehensive bibliographic search across multiple information sources, combining academic databases and grey literature repositories. The databases PubMed/MEDLINE, CINAHL Complete® and the Virtual Health Library (BVS) were systematically searched using controlled descriptors (Medical Subject Headings (MeSH), Health Sciences Descriptors (DeCS)) and free terms related to long-term care, policy, regulation, and ageing.

Controlled descriptors and free terms were combined using Boolean operators (“AND”, “OR”) and adapted for each database. An example of the preliminary search string used in PubMed and CINAHL Complete® was described in Table 1.

**Table 1:** Search strategy.

Search engine/ database/ repository	Search strategy	Data	Results
PubMed/Medline	(((("residential care facility"[Title] OR "long-term care facilit*"[Title] OR "long term care facilit*"[Title] OR "nursing home*"[Title] OR "Homes for the Aged"[Title]) OR ("Homes for the Aged"[MeSH Terms])) OR ("Nursing Homes"[MeSH Terms])) AND ((((("health policy"[Title] OR "healthcare policy"[Title] OR "public health policy"[Title] OR "strategic framework"[Title] OR legislation[Title] OR regulations[Title] OR guidelines[Title]) OR ("Health Policy"[MeSH Terms])) OR ("Facility Regulation and Control"[MeSH Terms])) OR ("Health Planning Guidelines"[MeSH Terms])) OR ("Practice Guidelines as Topic"[MeSH Terms]))) AND ((Aged[Title] OR Elderly[Title] OR "older adult*"[Title]) OR (Aged[MeSH Terms]))	12th August 2025	1 105
CINAHL Complete (EBSCO Host)	(TI ("residential care facility" OR "long-term care facilit*" OR "long term care facilit*" OR "nursing home*" OR “Homes for the Aged”) OR MH “Nursing Homes”) AND (TI ("health policy" OR "healthcare policy" OR "public health policy" OR "strategic framework" OR legislation OR regulation* OR guideline*) OR MH (“public policy” OR “health policy+” OR “Government Regulations+” OR “Practice Guidelines”)) AND (TI (Aged OR Elderly * OR elderly OR “older adult*”) OR MH Aged+)	12th August 2025	807

The search strategy combined controlled descriptors and free terms across CINAHL Complete®, MEDLINE, and BVS, complemented by searches on institutional websites and grey literature repositories such as the WHO, OECD, and national health agencies.

Reference lists of the included documents and pertinent reviews were also examined to identify further eligible sources. The retrieved records were imported into Rayyan® software platform to support screening and data management.

## Data collection and analysis

• Selection of Studies

The selection will take place in two stages:

1. Screening by title and abstract;
2. Full reading of the selected texts.

Two independent reviewers will carry out the process using the Rayyan® software platform. Conflicts will be resolved by consensus or by a third or fourth reviewer, blinding within Rayyan® software platform will be maintained throughout the process until consensus is achieved. The complete selection process will be illustrated in a PRISMA flow diagram, outlining the study identification and screening stages leading to the final sample.

• Data Extraction

During the data extraction process, a descriptive assessment of each study will be conducted to collect information relevant to the review question. This includes Type of text; Population represented; Setting or context; Stated allegiance or position of the text; Key conclusions and illustrative excerpts and Reviewer’s analytical notes, and stakeholders involved, will also be extracted. Data extraction will be independently performed by two reviewers, with any disagreements or uncertainties resolved by consultation with a third reviewer.

• Quality Appraisal

The methodological quality of the studies will be assessed using the JBI critical appraisal tool, tailored to the documents being analyzed. The JBI Checklist for Policy and Guideline Documents, will be applied. Two reviewers will apply the instruments independently, and any disagreements will be resolved through consultation with a third reviewer. The application of a systematic and rigorous JBI methodology will mitigate bias and strengthen the robustness and validity of the synthesized findings.

• Strategy for Data Synthesis

Data synthesis will be conducted in accordance with the JBI Manual for Evidence Synthesis, chapter on textual evidence reviews (policy documents). Considering that these are not quantitative studies, the analysis will be narrative, thematic and interpretative.

Initially, all included documents will be read in full for in-depth familiarization with the content and context. Next, the structured extraction of the data will be carried out through a table that will include information such as: Country or region of origin, text type, population represented, setting/ context, stated allegiance/position, conclusion and notes (Table 2).

**Table 2:** Draft data extraction instrument.

Types of Text	Population represented	Setting/Context	Stated allegiance/position	Conclusion	Reviewer`s conclusions and Notes
					

With the extracted data, on the main recommendations or policy measures and relevance for Residential care facility for Older People (ERPI), thematic codification will be carried out, identifying central concepts, arguments, proposals, gaps and recurrent descriptive evidence in the documents. The coding will be both inductive (emerging from the data) and deductive, based on the elements of the PICo question. The codes will be grouped into thematic categories that reflect the main emerging topics arising from the research.

The interpretative synthesis of these categories will seek to identify consensus, divergences and gaps in existing policies, as well as the evolution of institutional discourses over the last ten years. Where relevant, representative citations of the documents will be used to illustrate the analyses, ensuring fidelity to the original source (Table 3).

**Table 3:** Emerging themes of health policies in LTCF for older people.

Thematic Category	Subtopics	Sources that support	Representative Citations
			

The results will be presented in a structured manner, with narrative descriptions and comparative tables. Finally, a critical reflection will be carried out on the strength and weaknesses of textual evidence, its implications for health practice and for the management of ERPI, as well as suggestions for future investigations.

## Protocol validation

The protocol was validated through structured expert consultation to ensure methodological rigor, clarity, and relevance. Four experts were purposively selected based on predefined criteria, including: 1. possession of a doctoral degree in nursing or health sciences; 2. demonstrated experience in evidence synthesis or systematic reviews, with prior involvement in studies aligned with Joanna Briggs Institute (JBI) or PRISMA methodologies; and 3. recognized expertise in long-term care, gerontological nursing, or health policy research. Two of the experts had over ten years of professional and research experience in residential care settings for older adults, while all four had a solid background in methodological research and protocol development.

Validation was conducted in two stages. In the first stage, experts independently reviewed the protocol and provided written feedback regarding methodological coherence, feasibility, and alignment with JBI standards. In the second stage, an online consensus meeting was held to discuss and integrate the recommendations, leading to refinements in the eligibility criteria, data extraction framework, and synthesis strategy. This structured validation process is essential for producing a reliable and transparent synthesis of textual evidence on long-term care policies [11]. Furthermore, the use of predefined extraction and critical appraisal methods strengthens the consistency of results, particularly in contexts of normative and regulatory heterogeneity across countries.

In addition to expert consultation, the protocol underwent internal review by the research team and external validation through registration in the PROSPERO database, ensuring transparency, traceability, and methodological accountability. The use of standardized methodological frameworks and reporting guidelines is widely recognized as a key strategy to enhance transparency, consistency, and reproducibility in systematic reviews [12]. In the field of long-term care research, structured review approaches have also been shown to facilitate the systematic identification and comparison of policy-relevant domains, such as quality indicators and governance mechanisms across countries, particularly within OECD contexts [13].

Finally, the iterative development and refinement of this protocol were conducted in alignment with PRISMA-ScR guidelines and JBI standards. Any substantial amendments made during the review process will be comprehensively documented, safeguarding methodological integrity and reproducibility. Recent advances in qualitative evidence synthesis and policy document analysis underscore the importance of rigorous and transparent methodological frameworks, reinforcing the relevance of this systematic review for informing equitable and resilient long-term care policy development [[14], [15], [16]].

## Limitations

This review has some limitations that should be recognized. First, the synthesis relies on publicly available policy and regulatory documents, which may result in the exclusion of unpublished, internally used, or context-specific materials, potentially limiting the comprehensiveness of the evidence base. Second, the inclusion of documents from different countries and regulatory systems may introduce heterogeneity in terminology, scope, and governance structures, which can challenge direct comparison across jurisdictions.

Third, although the review follows the Joanna Briggs Institute methodology for textual evidence, interpretative synthesis inherently involves a degree of analytical subjectivity, particularly during thematic coding and categorization. To mitigate this limitation, data extraction and analysis will be conducted independently by two reviewers, with discrepancies resolved through consensus or third-party adjudication. Finally, language restrictions to English, Portuguese, and Spanish may lead to the exclusion of relevant policy documents published in other languages, introducing potential language bias.

## CRediT author statement

Conceptualization: F.B, M.K.A; Methodology: F.B, M.K.A; Validation: F.B., M.K.A, Z.C, and, C.M.V.; Resources: F.B., M.K.A, Z.C, and, C.M.V.; Writing original draft: F.B, M.K.A.; Writing review and editing: F.B., M.K.A, Z.C, and, C.M.V.; Supervision: Z.C, C.M.V. All authors have read and approved the final version of the manuscript.

## Related research article

None

## For a published article

None

## Declaration of competing interest

The authors declare that they have no known competing financial interests or personal relationships that could have appeared to influence the work reported in this paper.

## Data Availability

No data was used for the research described in the article.
